# Time lapse synchrotron IR chemical imaging for observing the acclimation of a single algal cell to CO_2_ treatment

**DOI:** 10.1038/s41598-021-92657-3

**Published:** 2021-06-24

**Authors:** Ghazal Azarfar, Ebrahim Aboualizadeh, Simona Ratti, Camilla Olivieri, Alessandra Norici, Michael J. Nasse, Mario Giordano, Carol J. Hirschmugl

**Affiliations:** 1 Beckman Institute, University of Illinois Urbana-Champaign, Urbana, 61801 USA; 2 Alcon Laboratories, Inc., Belmont, CA 94002 USA; 3 Dipartimento Scienze della Vita e dell’Ambiente, Universita’ Politecnica delle Marche, Ancona, AN Italy; 4 Karlsruhe Institute of Technology, Karlsruhe, Germany; 5 Institute of Microbiology, Academy of Sciences of the Czech Republic, Trebon, Czech Republic; 6 Department of Cell Biology and Molecular Genetics, University of Maryland, College Park, MD 20742 USA; 7 Department of Physics, University of Wisconsin-Milwaukee, Milwaukee, WI 53211 USA

**Keywords:** Biochemistry, Biological techniques, Biophysics, Cell biology, Molecular biology

## Abstract

Algae are the main primary producers in aquatic environments and therefore of fundamental importance for the global ecosystem. Mid-infrared (IR) microspectroscopy is a non-invasive tool that allows in principle studying chemical composition on a single-cell level. For a long time, however, mid-infrared (IR) imaging of living algal cells in an aqueous environment has been a challenge due to the strong IR absorption of water. In this study, we employed multi-beam synchrotron radiation to measure time-resolved IR hyperspectral images of individual *Thalassiosira weissflogii* cells in water in the course of acclimation to an abrupt change of CO_2_ availability (from 390 to 5000 ppm and vice versa) over 75 min. We used a previously developed algorithm to correct sinusoidal interference fringes from IR hyperspectral imaging data. After preprocessing and fringe correction of the hyperspectral data, principal component analysis (PCA) was performed to assess the spatial distribution of organic pools within the algal cells. Through the analysis of 200,000 spectra, we were able to identify compositional modifications associated with CO_2_ treatment. PCA revealed changes in the carbohydrate pool (1200–950 cm$^{-1}$), lipids (1740, 2852, 2922 cm$^{-1}$), and nucleic acid (1160 and 1201 cm$^{-1}$) as the major response of exposure to elevated CO_2_ concentrations. Our results show a local metabolism response to this external perturbation.

## Introduction

The adjustment of cellular responses to external changes is necessary for maintaining an efficient functional state in living cells and ensures fitness to the environment. Much of the understanding of cellular processes comes from experiments conducted at population level^1^. However, intrapopulation variability is increasingly emerging as a key aspect in the interaction between microorganisms and their habitat^2^. Single cell analysis is, however, a non-trivial endeavor, especially with respect to cell composition. Synchrotron-based Fourier transform infrared (FTIR) microspectroscopy is a non-invasive technique for chemical imaging of biological samples such as tissues and cells. A synchrotron FTIR microscope, equipped with a multi-element detector—focal plane array (FPA)—along with corresponding data processing techniques, can provide highly spatially resolved chemical information^3^. With the development of post-processing techniques to remove spectral distortions due to water absorption and the advent of microfluidic devices, live cell infrared microscopy became feasible. FTIR hyperspectral imaging provides information about the spatial distribution of important bio-chemical pools such as carbohydrates, lipids and proteins with diffraction-limited resolution^4–10^.

Algae constitute the main primary producers in aquatic environments. Their composition determines the quality of the organic matter that is then transferred to subsequent trophic levels. The understanding of their compositional responses to external perturbation is thus of utmost relevance. The production of new biomass in algae is based on the photosynthetic process. In current oceans, CO_2_ availability is insufficient to satisfy the requirements of photosynthetic organisms. In most cases, algae respond to this environmental challenge by activating energy-dependent CO_2_ concentrating mechanisms (CCMs). If ambient CO_2_ concentration increases to the point of allowing a diffusional flux towards the interior of the alga, CCM may be downregulated or turned off^11^. This may influence energy and C allocation^12,13^ in a species-specific manner^14^, with a strong dependence on the stoichiometry of the surrounding medium^13^ and light availability^15^. Compositional changes are also presumably linked to the exact mode of CCM downregulation: Memmola et al.^16^ showed that the inactivation of different CCM components results in different C allocation patterns. In very broad terms, one may expect that higher C availability leads to a size increase of those pools that mainly contain C and are poor of other macronutrients (i.e. carbohydrates and lipids^13,17^). Whether carbohydrates or lipids are selected as the pool where the excess C (relative to N or S, primarily) is allocated depends on species, cell size and energetic constraints^17^. Unfortunately, most C allocation studies on algae cultured at different CO_2_ concentrations have been conducted in time frames of days to weeks, due to the technical challenges of studying rapid compositional changes. Little is therefore known about short-term changes in the organic

**Figure 1:**
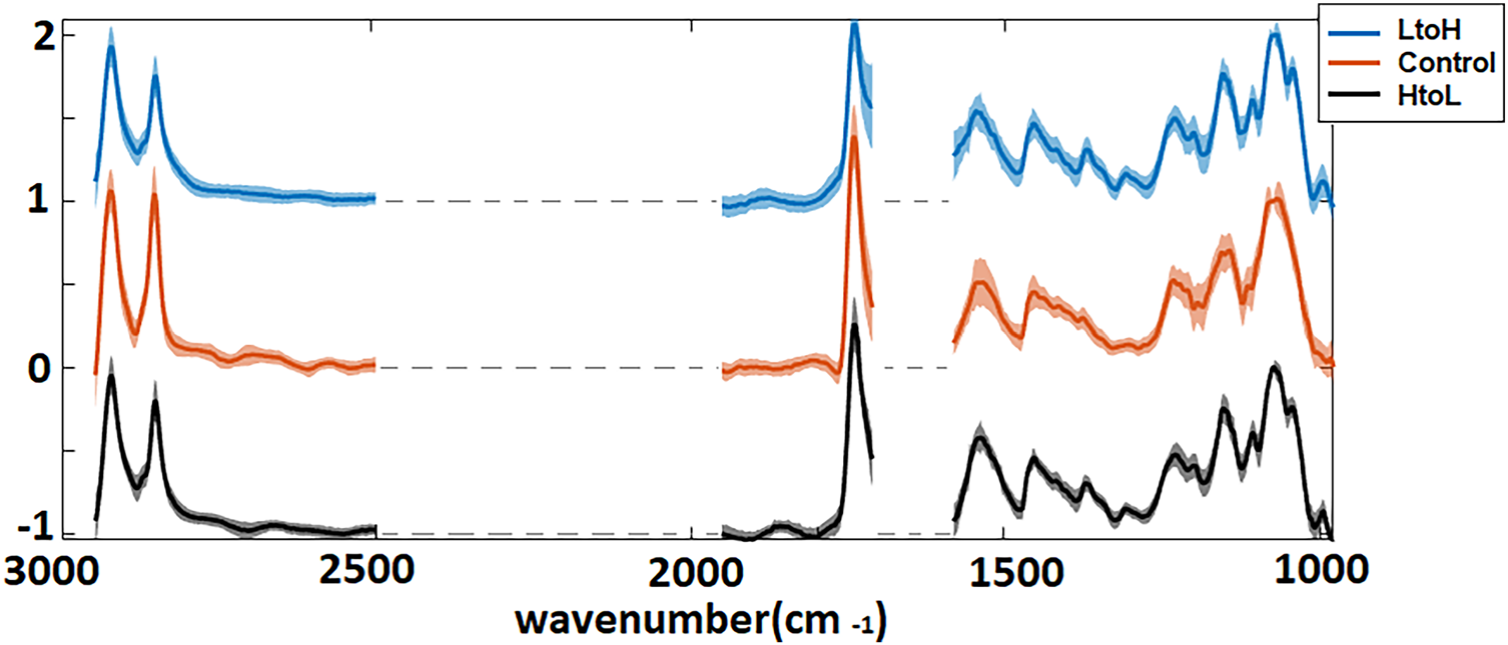
Average spectra of *Thalassiosira weissflogii* cells. The average spectra of a cell subject to a sudden change in CO_2_ availability and of a control cell are shown. “LtoH” refers to a change from low (390 ppm) to high (5000 ppm) CO_2_ concentration in the aqueous medium. “HtoL” refers to a high (5000 ppm) to low (390 ppm) CO_2_ concentration transition. The shadows of each curve indicate the variance of the averages.

composition of algae exposed to changing CO_2_. Also, averaging over an entire algae population may hide individual response patterns, which is why single cell studies are important.

Live cells must be kept in an aqueous environment in order to stay in their “normal” physiological state. Water is highly absorptive in the mid-infrared spectral region^18^, so, if FTIR is used for compositional studies, it is critical to design a microfluidic chamber with thin cross section reducing the absorption of mid-infrared light. In addition, the flotation and mobility of the cell in the chamber requires rapid measurements, resulting in low spectral and spatial resolution and signal to noise ratio (S/N)^19–21^. The thickness of the chamber must also be compatible with the high numerical aperture and small working distance of high resolution microscope objectives.

For this paper, a homemade microfluidic chamber, designed by Nasse et al.^22^, was employed to provide a controlled condition for FTIR imaging in aqueous environment. *Thalassiosira weissflogii* algal cells, cultured at ambient and elevated CO_2_ conditions, were transferred to the chamber for FTIR hyperspectral imaging. Single algal cells were monitored by an FTIR microscope for the span of 2 h at three different levels of CO_2_ availability. The collected four-dimensional hyperspectral cube had dimensions of ${64\times 64\times 1037\times 8}$, covering spatial, spectral and temporal domains.

The chamber was made of two sub-micrometer thick diamond windows. Since the wavelength of the light is comparable to the distance between the two diamond windows, multiple internal reflections occur. As a result, sinusoidal interference patterns, known as fringes, appear in the measured spectra. If the amplitude of the fringes is comparable to the amplitude of the absorption bands in the spectra, these fringes impact the chemical analysis. Various methods, such as interferogram editing, filtering, fringe fitting and subtraction from spectra have been used for correcting fringes from spectra^23–28^. Here, fringes were removed from the hyperspectral images based on the extended multiplicative signal correction (EMSC) method presented by Azarfar et al.^28^.

After preprocessing and fringe correction of the raw data, principal component analysis (PCA) was applied for data reduction purposes. To validate the results, we evaluated three replicates of low (390 ppm) to high (5000 ppm) CO_2_, high (5000 ppm) to low (390 ppm) CO_2_ concentration and control cells. The results show that the dominant spectral features were related to carbohydrates (1200–950 cm$^{-1}$) in response to changes in CO_2_ availability. The principal component (PC) score images suggest that the metabolism of the algal cells responded *locally* to the perturbation (see “Discussion”).

## Results

Figure 1 shows the average spectra of the three experimental conditions. They include two changes of the CO_2_ concentration, namely high-to-low and low-to-high, as well as a control. High-to-low refers to cells that were acclimated to high (5000 ppm) CO_2_ and then exposed to low (390 ppm) CO_2_ in the chamber at time 0 (experiment start). Low-to-high refers to cells that were acclimated to low (390 ppm) CO_2_ and then exposed to high (5000 ppm) CO_2_ at time 0. Control refers to cells that were acclimated to ambient (390 ppm) CO_2_ concentration without a change.

The average spectra of the algal cells typically explained more than 95% of the total absorption in the control and treated cells. Absorption bands of Si–O at ${\sim }$ 1075 cm$^{-1}$^29^, DNA–RNA at ${\sim }$ 1160 cm$^{-1}$^30,31^, P=O at ${\sim }$ 1240 cm$^{-1}$^29,31^, N–H bending associated with amide II for proteins at ${\sim }$ 1545 cm$^{-1}$^29,31^, and C=O stretch associated with lipids at ${\sim }$ 2852 cm$^{-1}$^31,32^ and ${\sim }$ 2922 cm$^{-1}$^31,32^ were observed in the average spectrum of the control and treated cells. The average spectra of the treated cells showed additional peaks: two peaks associated with polysaccharides at ${\sim }$ 1000 cm$^{-1}$^33^ and ${\sim }$ 1040 cm$^{-1}$^29^, as well as a peak at about ${\sim }$ 1201 cm$^{-1}$^30^, possibly attributable to DNA–RNA^30,31^. The peak assignments are summarized in Table 1.

**Figure 2:**
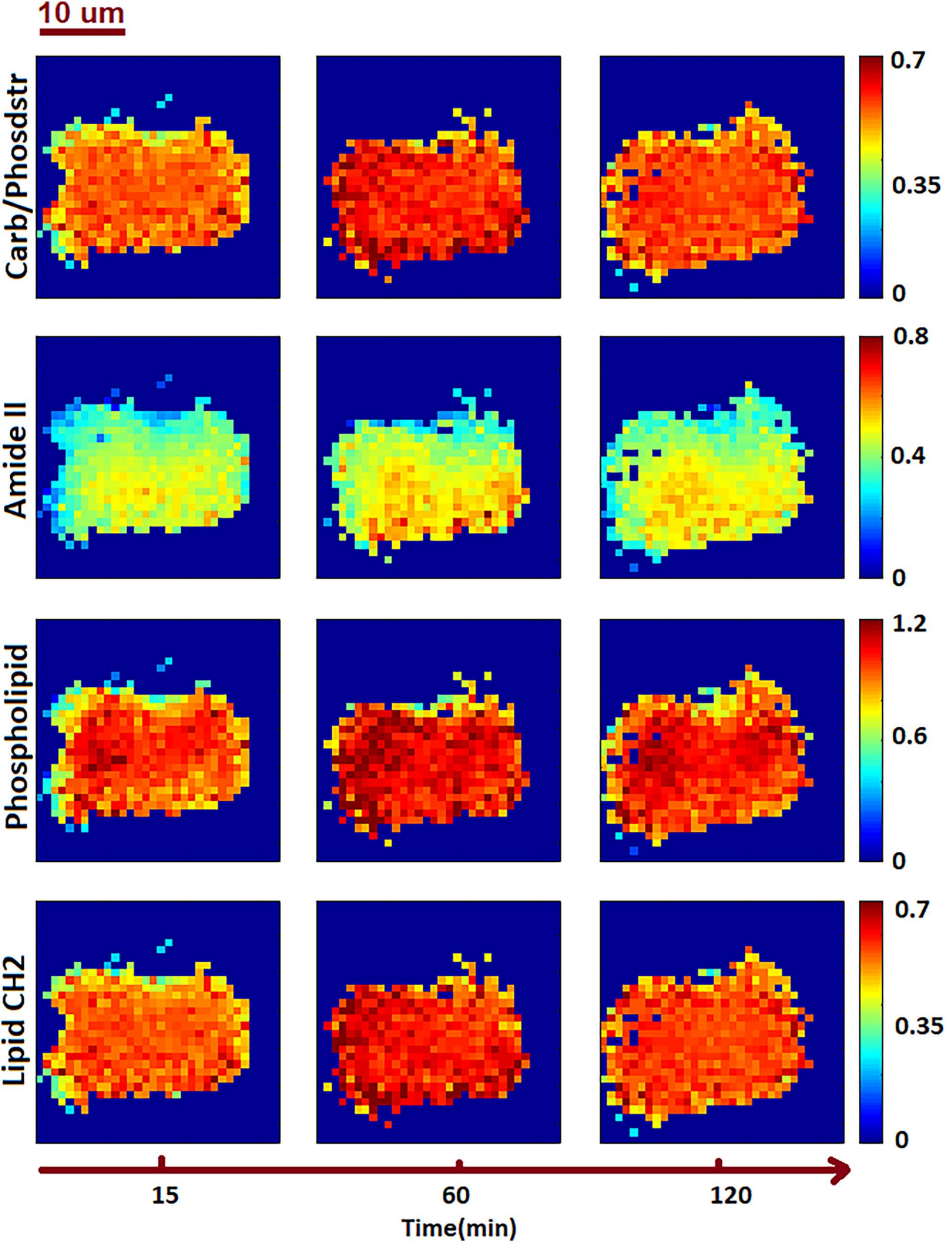
Areas under the chemical bands. Spatial changes of macromolecules as a function of time for the control condition. Integrated images of carbohydrate/phosphodiester (1005–1280 cm$^{-1}$), amide II (1500–1570 cm$^{-1}$), phospholipids (1720–1753 cm$^{-1}$) and lipid (2821–2943 cm$^{-1}$) are shown.

Pixels that contain algal cell absorption signature are normalized at Si–O peak at 1075 cm$^{-1}$, and then they are fringe corrected^28^. In case that the fringe correction algorithm failed for correcting a pixel, the spectrum of the pixel was replaced by zeros. After fringe removal, the areas under the spectral range associated with carbohydrate/phosphodiester (1005–1280 cm$^{-1}$), amide II (1500–1570 cm$^{-1}$), phospholipids (1720–1753 cm$^{-1}$) and lipid (2821–2943 cm$^{-1}$) are calculated. Figure 2 shows the peak areas as a function of time after fringe correction. Columns of the Fig.  2 correspond to the time of the measurement, the first column represents the first measurement at time equal to 15 min, and the last column represents the last measurement at time equal to 120 min. The dark pixels close to the borders of the cell have a strong fringe contribution. The fringe correction algorithm did not correct these pixels, and hence these pixels are replaced with zero. Figure 2 shows a slight variation in the spatial distribution of the cell as a function time. Applying PCA to the data shown in Fig. 2 reveals the response of the algal cell to CO_2_ availability.

**Table 1:** Infrared absorbance peak assignment for *Thalassiosira weissflogii*.

Functional group	Band assignment (cm$^{-1}$)
C–OC/polysaccharides	$\sim \,1000$, $\sim \,1040$, $\sim \,1080$
DNA–RNA	$\sim \,1160$, $\sim \,1201$
P=O/phospholipids	$\sim \,1240$
N–H bending/amide II	$\sim \,1545$
Lipid and fatty acid C=O/CH$_{2}$	$\sim \,1740$
Lipid/CH$_{2}$	$\sim \,2852$, $\sim \,2922$

**Figure 3:**
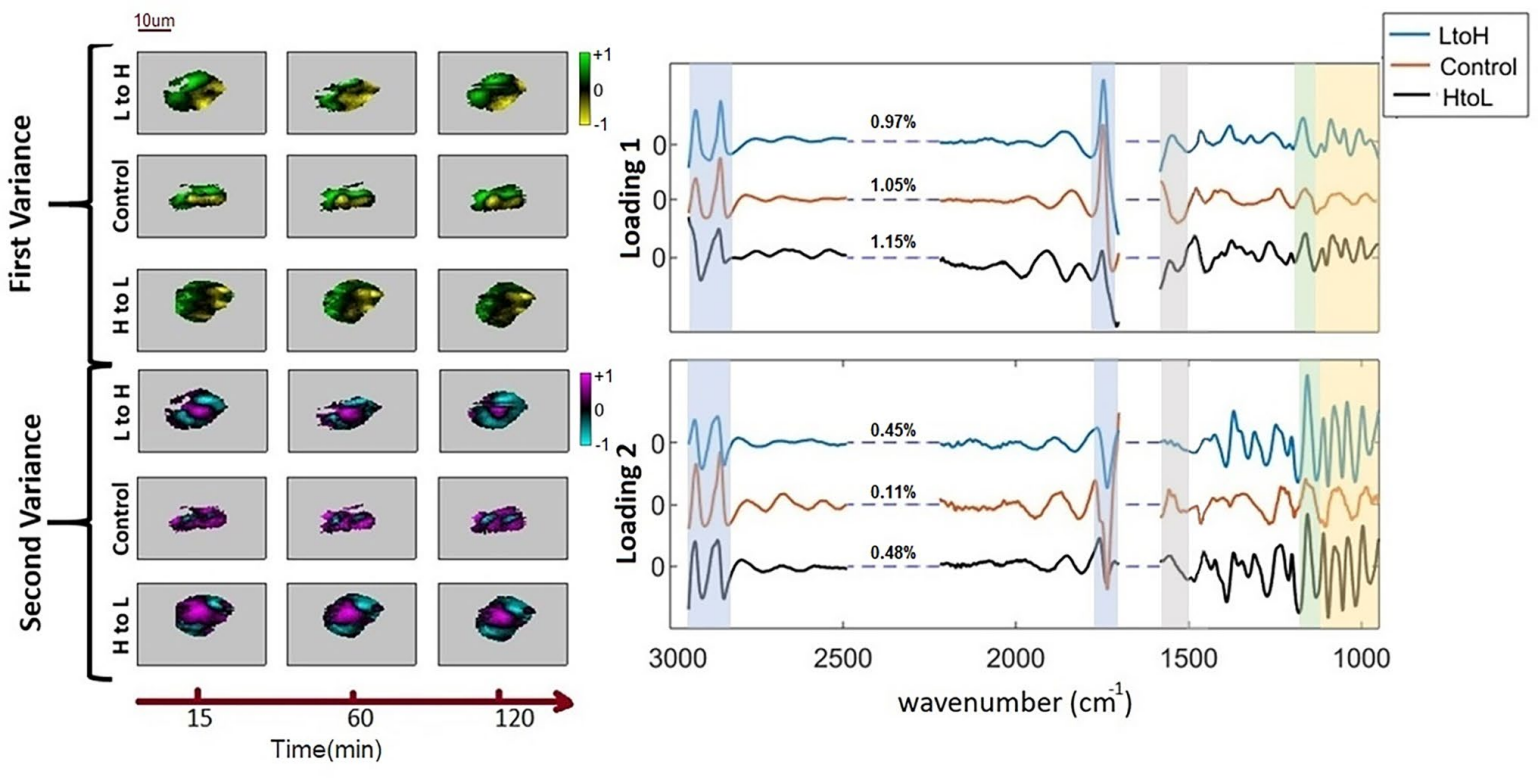
Correlation between spectral features and spatial distribution. Principal component analysis was done for each algal cell separately. Left: PC score images over time (gray indicates the background); right: PC loadings. The percentages on each loading shows the average of the percentage of the total variance explained by each principal component along the replicates of the experiment. Colored vertical bands indicate chemical compounds: orange: carbohydrate, green: DNA–RNA, gray: amide II, blue: lipids; “LtoH” indicates the low (390 ppm CO_2_) to high (5000 ppm CO_2_) treatment. “HtoL” indicates the high (5000 ppm CO_2_) to low (390 ppm CO_2_) treatment.

Figure 3 shows the first and second variances and their corresponding score images for the three conditions, control, low-to-high and high-to-low. For all PC score images, gray corresponds to the background pixels of the image. The rest of the pixels are categorized into positive, neutral, or negative PC scores. First and second variance images have both positive and negative scores. A pixel has a negative (positive) score if the corresponding spectral variations are dominated by negative (positive) variations of the principal component loadings.

Thus, PC score images show local spectral variations and can therefore indicate the direction of maximum spatial variations in the spectra. For example, the first variance of all the treatments and control samples in Fig. 3 (yellow—negative, black—neutral, and green—positive) shows a sharp change in the lipid band, mainly along the vertical direction. One half of the cell is predominately green, and the other half is predominately yellow. Notably, all cells show the same left-right split, independent of cell orientation, most likely due to the direction of the medium flow in the microfluidic chamber. The second variation images (cyan—negative, black—neutral, purple—positive) reveal a wider variety of features. Columns of the score images are associated with the time of the measurement. The first column shown corresponds to the measurement at time 15 min, the second column to the measurement at time 60 min, and the third column to the measurement at time 120 min.

The first and second variances represent less than 2% of the chemical contributions in the spectra, and the remaining PCs are dominated by noise (${\sim }$ 3%). The largest variations were observed for the lipids and carbohydrates. The reproducibility of the result was tested by investigating six sets of data, three replicates of control, and three replicates of treated cells (low-to-high and high-to-low CO_2_ both). Typically, PCs are classified based on the percentage contribution of each PC to the overall composition. To facilitate comparisons between control and treated cells, PCs are here classified based on the similarity of their images, evaluated from score plots and spectral loadings.

First variance images and loadings, which represent approximately 1% of the composition of the total cells, confirm the impact of the treatment on the carbohydrate pool, but also show changes in lipids (at 1740 cm$^{-1}$, 2852 cm$^{-1}$, and 2922 cm$^{-1}$).

The second variance is representative of the strongest variation, after lipids, over the hyperspectral data set and explains less than 0.5% of the total absorption due to the composition of the cell.

**Figure 4:**
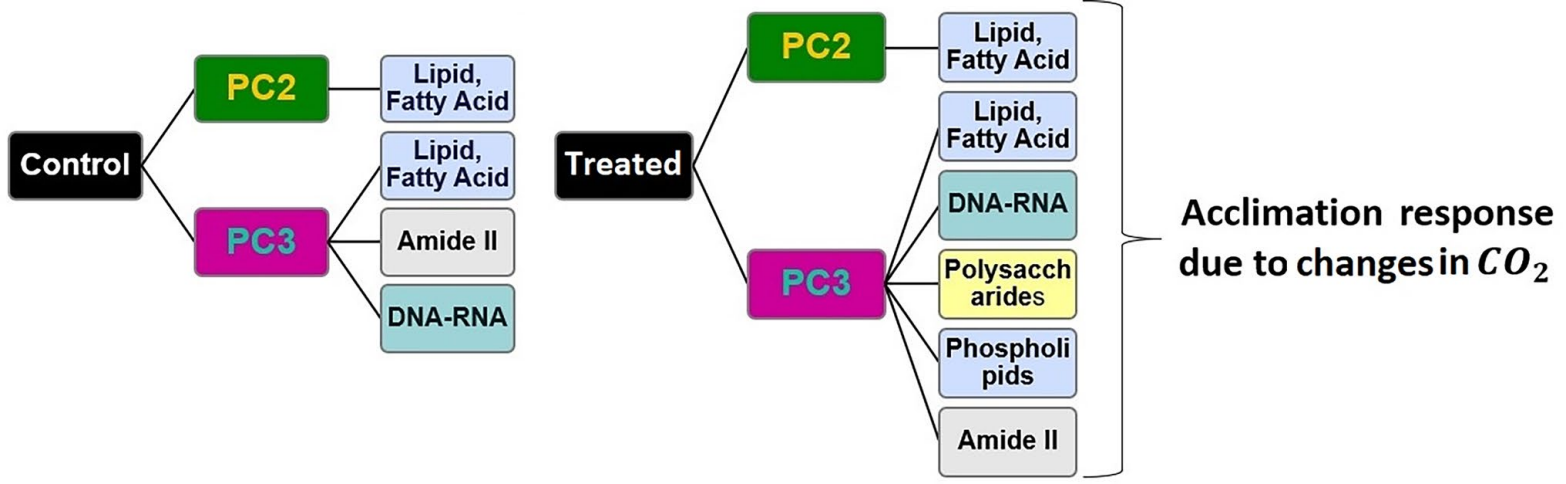
Dominant macromolecular changes. Chemically distinct molecules observed in each principal component (PC). The first variance is considered as PC2, and the second variance is PC3.

Besides lipids, peaks at ${\sim }$ 1160 cm$^{-1}$ and ${\sim }$ 1545 cm$^{-1}$ are present in the second variance of the controls, while bands at ${\sim }$ 1000 cm$^{-1}$, ${\sim }$ 1040 cm$^{-1}$, ${\sim }$ 1080 cm$^{-1}$, ${\sim }$ 1160 cm$^{-1}$, ${\sim }$ 1201 cm$^{-1}$, and ${\sim }$ 1240 cm$^{-1}$ were observed only in the treated cells. Hence, the second variance spectrum of treated cells shows variation in carbohydrate, lipids and nucleic acid. While the second variance features the lipids as the dominant variation in the control data, bands due to polysaccharides and RNA–DNA become prominent in the treated data. The polysaccharide increase may be explained as above. A higher RNA content may be associated with a growth stimulation at elevated CO_2_. The dominant peaks in the PC variances are summarized in Fig. 4 for treated and control conditions.

**Figure 5:**
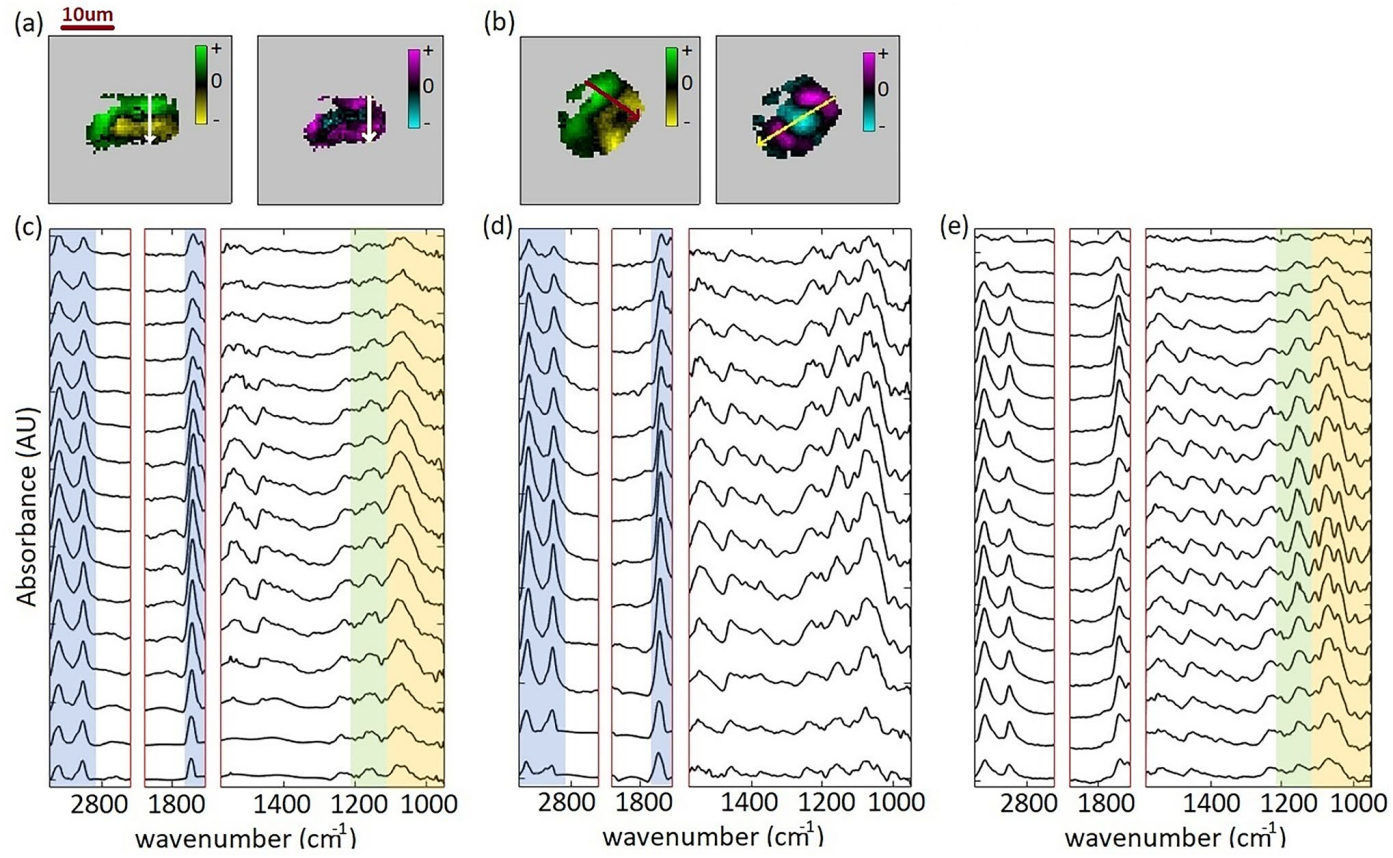
Principal component variance images and stack of spectra, 15 min after experiment start. Top: PC Score images of the first measurements show the intracell variance. Here arrows indicate the direction of the maximum spectral variations in the variance images. Below: stack of spectra (top to bottom) along the arrows. (**a**) First and second variance of the control cell. (**b**) First and second of the variance of the treated cell with low-to-high CO_2_ concentration. (**c**) Stack of the spectra along the white arrow. (**d**) Stack of the spectra along the red arrow. (**e**) Stack of the spectra along the yellow arrow.

The variance images shown in Fig. 3 show the direction of maximum spectral changes across the cell as described above. Stacks of spectra along lines from positive to negative regions of the variance images reveal the distribution of spectral variations in the cell (Fig. 5). Figure 5a,b show the score images of the control and treated (low to high) cell respectively. The apparent size of the cells in the first and second variance images shown in Fig. 5a,b is changing, since the number of zeros in the first and second variance score images are different. The stack of spectra for the control cell is shown in Fig. 5a. The spatial distribution of the first and second variance spectra for the control cell, and a path through the cell from a positive to a negative edge are shown. This stack of spectra from the top to the bottom of the control cell provides information about the spatial variations of lipid, DNA–RNA and carbohydrates. This is related to the similarity of the first and second variance images of the control cell.

Figure 5b shows a stack of spectra across the arrow sketched in the first variance image of the treated cell (acclimated to 390 ppm CO_2_, then exposed to an elevated CO_2_ concentration). The stacked spectra demonstrate lipid changes in the treated cell. Figure 5c shows the distribution of the carbohydrates and DNA–RNA peaks of the same cell.

Maximum fluctuations in the second variance occurred at 1740 cm$^{-1}$, 2852 cm$^{-1}$, and 2922 cm$^{-1}$. Spectral regions associated with these peaks are highlighted with blue vertical bands (Fig. 5a,b). Notice that similar changes in the lipid peaks occur from the green toward black and toward the yellow regions of the cells for both control and treated cells. The main differences between the stacks of spectra in Fig. 5a,c are highlighted in green (1201 and 1160 cm$^{-1}$, 1126–1213 cm$^{-1}$) and orange (1000, 1040 cm$^{-1}$, from 950 to 1126 cm$^{-1}$). Note that for both control and treated cells, the stack of spectra indicate similar trends for functional groups.

## Discussion

The sensitivity of aquatic photo-autotrophs to CO_2_ certainly varies with species and the mechanisms by which cells perceive changes in the concentration of dissolved inorganic carbon (DIC). In the case of neritic species, where the environment changes markedly in CO_2_ concentration and photon flux density, the CCM may be regulated using a CO_2_-sensing mechanism. This type of sensing mechanism may be an adaptation to a habitat; that may be advantageous to the survival of an alga to respond to changes in CO_2_ concentration^34^.

The time required to fully express DIC transport has been found to be 3–6 h in freshwater algae depending on the species. Few reports are available on time course of acclimation of high CO_2_-grown marine. For diatoms, the literature shows that they can induce DIC transport with a 2–4 h lag period and that the alga is relatively slow to develop high-affinity photosynthesis when exposed to CO_2_ limiting conditions^34^.

The growth and photosynthetic activity of diatom *Thalassiosira weissfloggii* has been reported in the time span of days^35^, and to author knowledge not much is reported about its short-term acclimation to CO_2_ treatment. We evaluated the distribution and time resolved modifications of organic cell pools under controlled hydrated environment in viable algal cells.

A former paper published by several of authors^28^ along with the deliberate analysis provided in Appendix B, indicated that the effects of fringe correction method^28^ and the IR beam symmetry^4^ are minimal. To confirm that the algal cells remain alive in the environmental chamber measurements were extended to more than 9 h. The measurements of the treated cell (shown in Appendix C Figure S5) is indicative of the preservation of the cellular architecture for at least 5 h long, suggesting that cytoskeleton-bound enzymes provide a dynamic network, controlling transport and localization within the algal cells^36^.

The average spectra (shown in Fig. 1) of the control and treated cell show a difference mainly in the carbohydrate spectral region (between 1200 and 950 cm$^{-1}$), compatible with an increased accumulation of stored carbohydrates, resulting from an imbalance in the elemental stoichiometry of treated cells (i.e. the ratio of C to other nutrients increased when cells are cultured at elevated CO_2_). This analysis provides a gross overview of the changes to the chemistry of the treated cells.

The statistically driven PCA is more useful to evaluate the subtle changes in the cells due to dissolved CO_2_ conditions. The PCA includes hyperspectral images of one cell over the span of 2 h (8 measurements), implying that the variation is with respect to the average spectrum in time. Figure 3 show slight variations in the color distributions as a function of time. When the colors range across the scales (yellow to black to green or Blue to black to purple), they correspond to the changing sign for the score images. One trend that is observed in data for the treated samples, from earlier to later times in these series for first and second variances show an increasing number of pixels with colors associated with negative signs (yellow for the first variance and blue for the second variance). These statistically robust temporal variations are indicative of the subtle variations due to treating the cells. In parallel, with the scores, spectroscopic signatures provide insight into the chemical variations that are correlated with the temporal–spatial variations.

The first variance score images and loadings represent approximately 1% of the absorption due to the composition of the cell. When comparing the magnitude of this for control versus treated cells, there is little to no difference in the percentage of statistical changes or visual modification of score images in the first variance score images. Subtle spectral variations in the loadings of the control versus treated cells and the gradients from left to right side of all the cells for the first variance images (including the results shown in supplementary materials (appendix A and B)) is always green (positive), while the right side of the cell is yellow (negative). Since this is a common response to the stimuli for both the treated and control cells, a common impact must be leading to this variance. The authors suggest that the mechanical stress of the flow cell could impact the morphogenesis of developing single cells that depends on the microtubule cytoskeleton, which is regulated by mechanical stress^37,38^. We can relate the visual left and right split of the cell in the first variance images to two potential aspect of the experiment which is common for the control and treated cells: the mechanical stress from the microfluidic chamber and the direction of the flow of the media and nutrients that keep the cells viable throughout the experiment. An interesting observation from the stacks of spectra along the direction of changes in the first variance images show that there is an increase in lipid storage from the left side of the cell. The authors suggest that this is a chemical response to the mechanical stress.

There are differences in the second variance of the treated and control cells, differences in their percentage of the statistical changes, spectroscopic variations of the loadings and visual modification of scores. The percentage of the variations in treated cells are more than four times larger than the control cell and are associate with carbon-based macromolecular pools-explicitly carbohydrates, and DNA–RNA. Since the only controlled difference between the control and treated cells is the addition or reduction of dissolved CO_2_ in the medium for the LtoH and HtoL. Hence, the second variance is representative of the chemical response of the cell to modification of the medium. The polysaccharide increase is likely due to the change in the metabolism in response to CO_2_ changes, and is regulated by CCM, and a higher RNA content may be associated with the stimulation of growth at elevated CO_2_. The measured batch culture growth rates of 0.4 $\upmu \hbox {mol L}^{-1}\,\hbox {day}^{-1}$, and $0.55\,\upmu \hbox {mol}$ L$^{-1}$ day$^{-1}$ for CO_2_ concentrations of 390 and 5000 ppm, respectively, are also consistent with our findings for a single cell.

The findings summarized in Fig. 4 indicate a change in the response of the treated cells versus the control cells. Intrestingly, the PCA of the “LtoH” and “HtoL” conditions are similar to one another, even though the designed experiments change dissolved CO_2_ concentrations in opposite directions. Since the response of the cells is independent of the trend in the concentration change the authors conclude that the *Thalassiosira weissflogii* respond with a fast response that is dependent only on the change in the dissolved CO_2_ concentrations. Thus, the short-term acclimation process of the algal cell is responding only to the change in CO_2_ concentration not the trend (see Appendix D).

The spectroscopic data show varying intensities for carbohydrates and lipids over the time series and can be attributed to the following cellular processes. The peak in intensity of the carbohydrates (shown in Fig. 2) at time equal 60 min is followed by a decrease in the carbohydrates and may be explained by the three concurrent processes of photosynthesis, gluconeogenesis and glycolysis^39^. In addition, the cell has not yet divided, and still requires new membrane lipids. The increasing lipids could be due to fatty acids synthesized in the chloroplast that may be processed to generate membrane lipids, or stored as triacylglycerols^40^ resulting in an increase in phospholipid and lipids.

Responses of the carbon metabolism pathways are variable between different studies even for cell cultures, making general observations on these responses difficult^40^. In this work we show the ability to extract subtle IR spectral variation, and therefore chemical variation, for single living cells in varying aqueous environments. It is critical to also capture the wide range of individual responses in addition to capturing population level responses, which is the demonstrated value of the present experiments. Drawing strong conclusions for the variations among the population level are only possible with carrying out more studies in future.

## Methods

### Microalgae growth conditions

The centric diatom *Thalassiosira weissflogii* was cultured semi-continuously in AMCONA medium^41^ at constant irradiance (100 $\upmu \hbox {L}$ m$^{-2}$ s$^{-1}$, PAR) and at 20 $^\circ$C. All cultures were maintained in 250 mL Erlenmeyer flasks in a temperature-controlled growth chamber (SANYO Mir 154). The control, and the low-to-high cultures were continuously bubbled with filter-sterilized atmospheric air containing 390 ppm CO_2_, and the high-to-low treated cells were bubbled with filter-sterilized atmospheric air with elevated CO_2_ concentration of 5000 ppm. All the experiments were carried out on cells in exponential growth phase.

### Controlled aqueous environment

A demountable microfluidic chamber was used to provide a controlled aqueous environment with the same condition as the original cultures, for single cell imaging (exception: case III). The microfluidic device was made of two parallel diamond windows (typical thickness of 0.4–0.8$\,\upmu \hbox {m}$), held apart by a spacer (PIKE Technologies, Madison, WI—typical thickness of $15\,\upmu \hbox {m}$). A syringe-based push/pull pump was used to drive the liquid through the chamber with a flow rate of $10\,\upmu \hbox {m}$ L/min^24^. The water layer thickness in the chamber was between $20\,\hbox {and} \,25\,\upmu \hbox {m}$, thus different from the $15\,\upmu \hbox {m}$, nominal thickness of the spacer. This was the result of bulging of the thin diamond membranes due to water pressure.

### FTIR hyperspectral imaging

We used an FTIR Bruker Hyperion 3000 microspectrometer as an imaging system. It was equipped with a focal plane array (FPA) detector ($64\times 64$ pixels) and coupled to a synchrotron-based IR source (using the Infrared Environmental Imaging beamline at the Synchrotron Radiation Center)^4,41^. The sample was illuminated by a $20\times$ (0.6 NA) Schwarzschild condenser. Each FPA pixel is imaged by a $74\times$ Schwarzschild objective (0.6 NA) and corresponds to a sample area of $0.54\times 0.54$ $\upmu \hbox {m}$^2^.

To measure the short-term chemical response of the algae, cells were treated with two environmental conditions, low-to-high and high-to-low. The cultures acclimated to low/high (390 ppm/500 ppm) CO_2_ were transferred directly from the culture to the environmental chamber with an automatic pipette. The volume of the culture transferred was  10 $\upmu \hbox {L}$. The environmental chamber was filled with $5\times 10$ $\upmu \hbox {L}$ of fresh growth medium. Subsequently, the system was bubbled with air enriched with high/low (5000 ppm/390 ppm) CO_2_. A control experiment was carried out by using atmospheric air (390 ppm CO_2_). After the cells settled down in the chamber, an individual cell was single out and followed throughout the experiment duration.

The FTIR spectral images were collected every 15 min over the span of 2 h (8 measurements). Three replicates of the data for the control and three replicates of the data for the treatment were collected. The collected data is publicly available from UWM digital commons.

### Preprocessing

To remove the effect of total absorption of the water, the hyperspectral cubes were truncated below 948 cm$^{-1}$ and above 2946 cm$^{-1}$. Then the spectra were smoothed by a Savitzky–Golay filter of order two with 9 points. Since there are no relevant chemical information in the regions of the image where there is no algal cell, the images were segmented into cell and background area by means of Otsu’s method^42^. Next, the frequency of fringes observed in each individual spectrum was determined by calculating the Fast Fourier transform (FFT) of the absorbance spectrum in the spectral range of 1766–2790 cm$^{-1}$. The fringes are modeled as a component of the extended multiplicative signal correction (EMSC) method, and the baseline effects and fringes are removed from the spectra by means of the EMSC^28^.

### Dimensionality reduction

The measured data is a 4-dimensional cube of information ${(x, y, \nu , t)}$, where *x* and *y* indicate the 2 spatial dimensions, ${\nu }$ is the wavenumber of the chemical image and *t* is the measurement time. Approximately 5000 spectra were extracted from each measurement (total: 200,000 spectra). PCA was used for dimensionality reduction. PCA can be considered as a mapping from the measurement coordinate system ${(x, y, \nu , t)}$ to a new coordinate system made of PCs. The PCs are a set of uncorrelated orthogonal bases and are considered as the coordinates of the new system. When the data cube is not scaled, the first PC is the average of the spectra, the second PC indicates the largest possible variance from the average as a function of wavenumber. Each succeeding component has the next highest variance possible under the constraint that it is orthogonal to the preceding component^43^. Projecting the data cube into this new coordinate system results in score images, that show the spatial distribution of the variance.

For the analysis, we used MATLAB Statistics and Machine Learning Toolbox release 2015a. MATLAB centers the data and uses the singular value decomposition (SVD) algorithm to perform PCA. In our measurements, only the first two orthogonal variances contain spectral features that provide chemical information; the rest are dominated by noise. The data collected from each cell -the 8 measurements over the span of 2 h-was considered as an input for the PCA, meaning the PCA was done independently for each cell. In order to remove the impact of water and CO_2_, only the wavenumber ranges of 2946–2489 cm$^{-1}$, 2219–1702 cm$^{-1}$, and 1581–950 cm$^{-1}$ were considered as PCA variables.

## Supplementary Information

Supplementary Information 1
