# Supplementary material for: Time lapse synchrotron IR chemical imaging for observing the acclimation of a single algal cell to CO2 treatment
Source: Sci Rep. 2021 Jun 24;11:13246. doi: 10.1038/s41598-021-92657-3 (PMC8225881; doi:10.1038/s41598-021-92657-3)
Supplement: Supplementary file 1 — Supplementary Information 1 [file 41598_2021_92657_MOESM1_ESM.pdf]

Supplementary Information for

Time Lapse Synchrotron IR Chemical Imaging for Observing the Acclimation of a Single Algal Cell to CO_2 Treatment

Ghazal Azarfar[1], Ebrahim Aboualizadeh[2], Simona Ratti[3], Camilla Olivieri[3], Alessandra Norici[3], Michael J. Nasse[4], Mario Giordano[3,5,6], and Carol J. Hirschmugl[7,*]

[1]University of Illinois Urbana-Champaign, Beckman Institute, Urbana, 61801, USA
[2]Alcon Company, NY 14627, USA
[3]Universita' Politecnica delle Marche, Dipartimento Scienze della Vita e dell'Ambiente, Ancona AN, Italy
[4]Karlsruhe Institute of Technology, Karlsruhe, Germany
[5]Institute of Microbiology, Academy of Sciences of the Czech Republic, Trebon, Czech Republic
[6]Department of Cell Biology and Molecular Genetics, University of Maryland, College Park, MD 20742, USA
[7]University of Wisconsin-Milwaukee, Department of Physics, Milwaukee, WI 53211, USA
[*]cjhirsch@uwm.edu

Appendix A

To confirm the consistency of the PCA results, the results of the 3 replicates of the control and stressed experiments (cells habituated to 390 ppm CO_2 and exposed to a flow with 5000 ppm CO_2 in the chamber) are provided in Figure S 1 to S 3. To increase the contrast of the cells from the black background, the surrounding areas of the cell are greyed out.

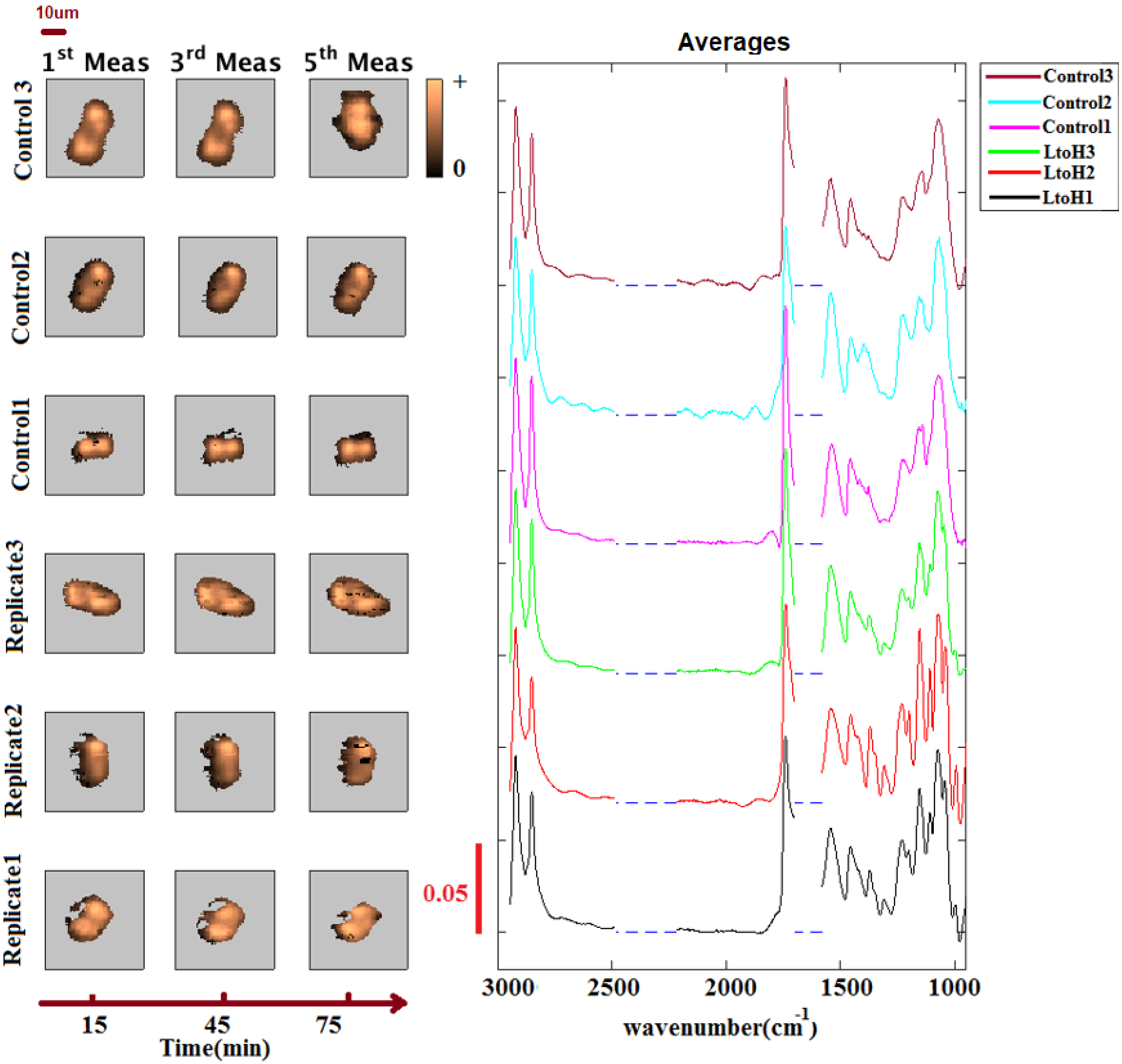

Figure S 1. PC1 (Average) PC1 loadings (Average), and score images for 3 replicated of treated, and 3 replicates of the control condition. PC score images, and loadings are in good agreement from one measurement to another. The first three rows of the images are control replicates, and the second three row of the images are the treated ones (from 390 ppm to 5000 ppm CO_2).

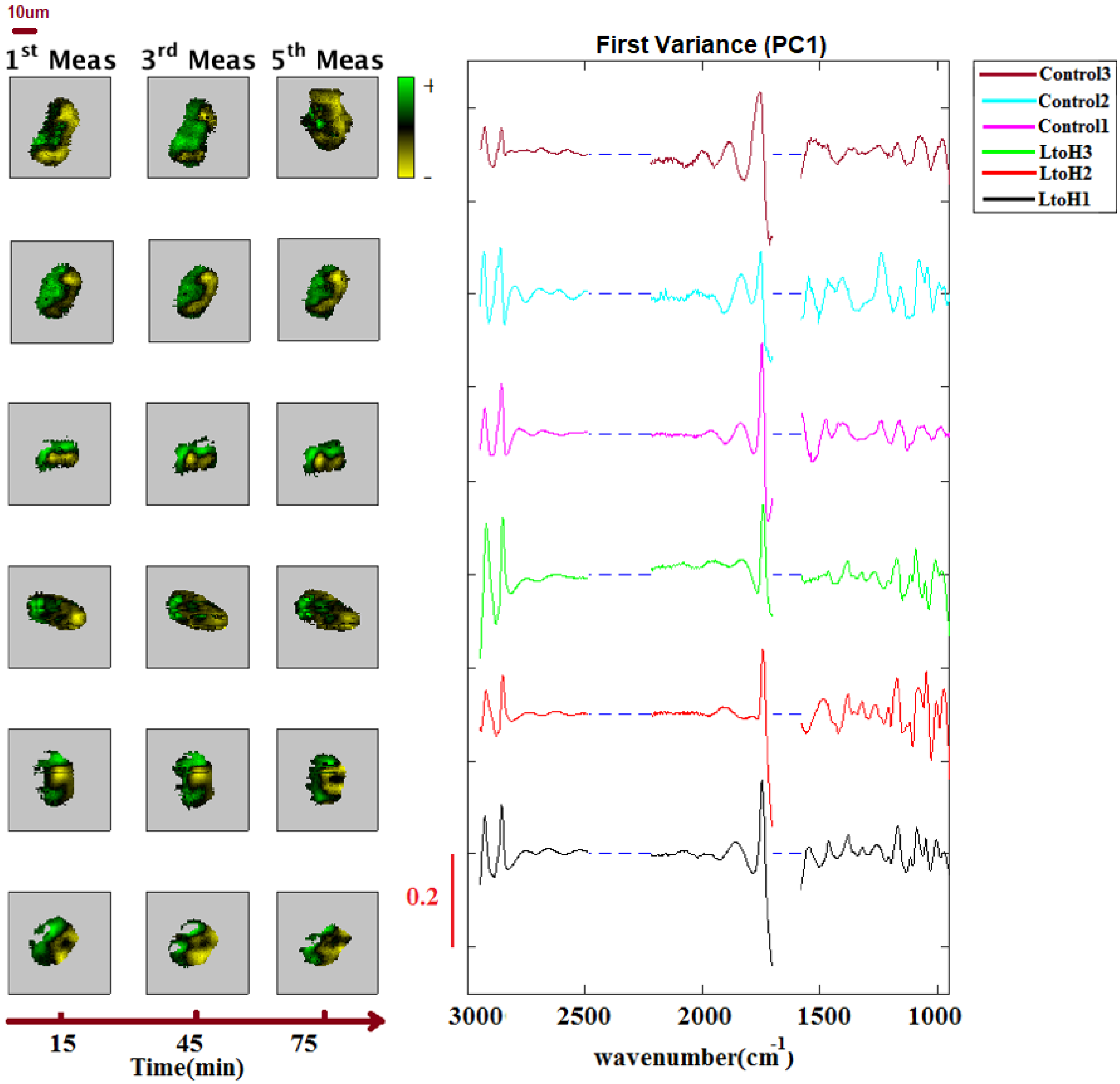

Figure S 2. PC2 (First Variance) PC2 loadings, and score images for 3 replicates of control, and 3 replicates of treated cells. PC score images, and loadings are in good agreement from one measurement to another. The first three rows of the images are control replicates, and the second three row of the images are the treated (from 390ppm to 5000 ppm CO_2) replicates.

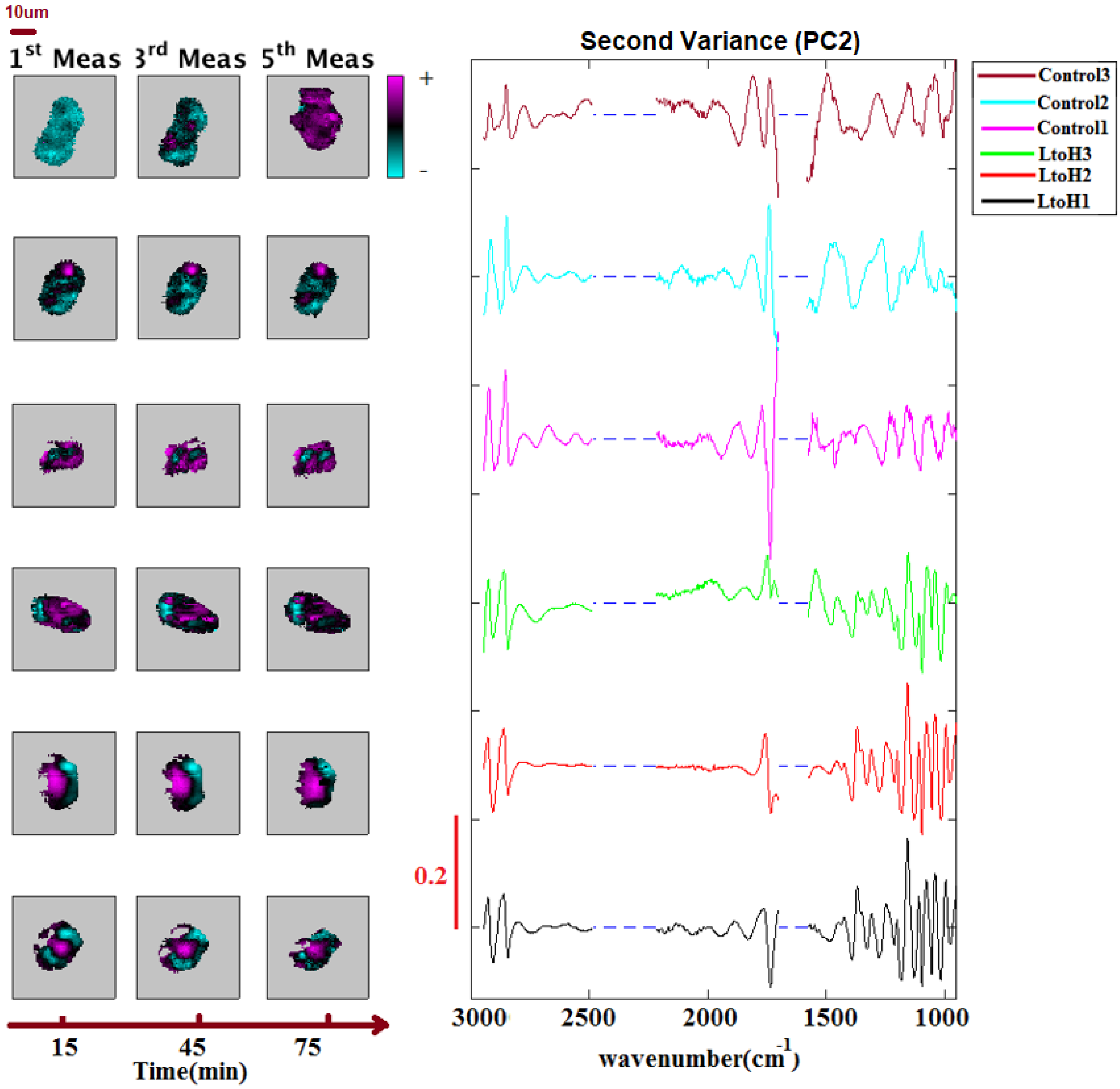

Figure S 3. PC3 (Second Variance) PC3 loadings, and score images for 3 replicated of control, and 3 replicates of treated cells. PC score images, and loadings are in good agreement from one measurement to another. The first 3 rows of the images are control replicates, and the second 3 rows of the images are treated (from 390 ppm to 5000 ppm CO_2) replicates.

Appendix B

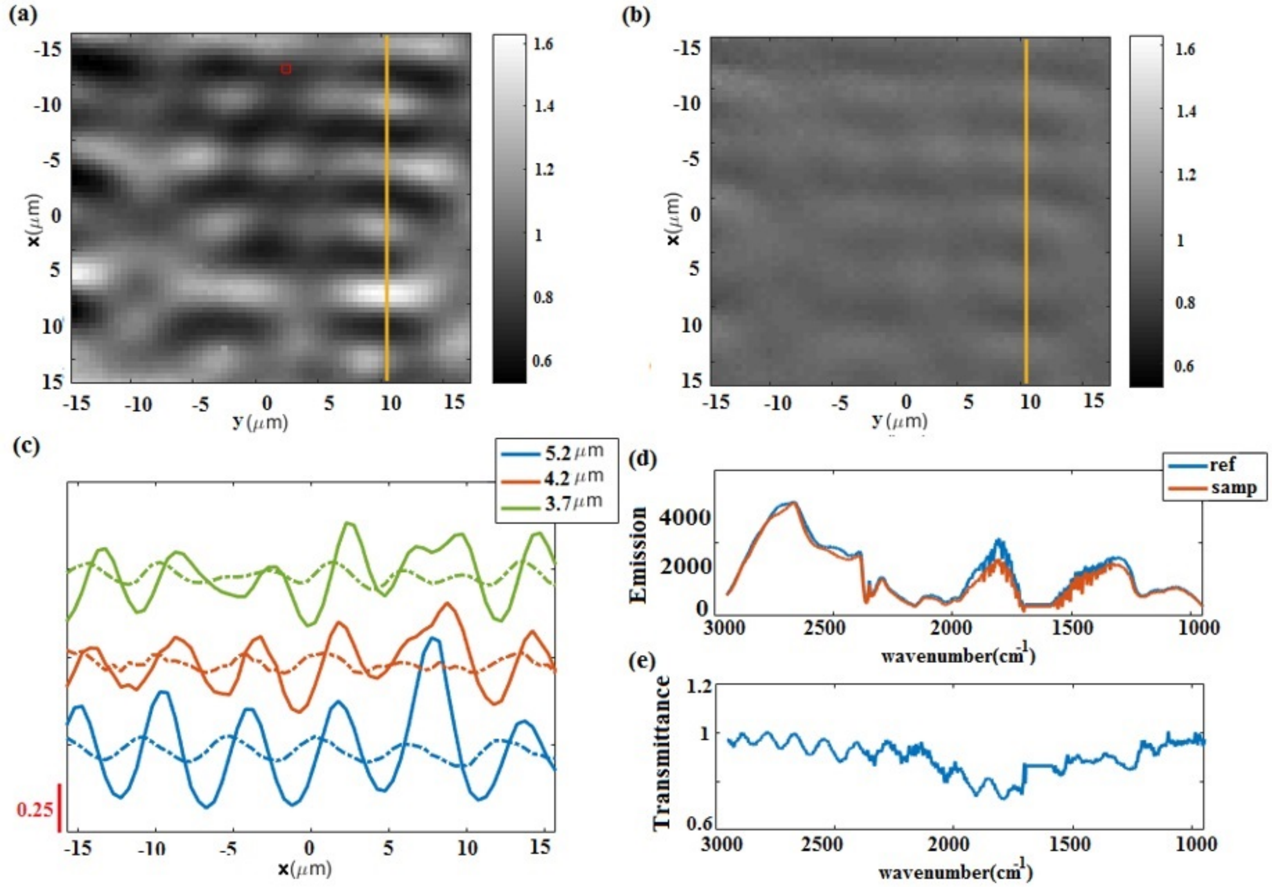

Figure S 4. Imaging in aqueous environment (a) emission image at sample (position 2) divided by emission image at reference (position 1), at 5.2 μm wavelength. (b) first measurement of emission image at sample (position 2) divided by second measurement of emission image at sample (position 2), at 5.2 μm wavelength. (c) Line scans through the transmittance images at yellow line, pallet a: solid line, pallet b: dashed line. (d) emission spectrum of the reference, and sample at the pixels identified with red color. (e)Transmittance spectra at red pixel.

Infrared transmission hyperspectral data cubes are measured by collecting two sets of images, one reference or background hyperspectral data cube, and one sample data cube. Reference data cubes are taken from a location as close to the algal cell as possible. The second set of data cubes (sample data cubes) are taken from a region including an algal cell. Dividing a sample data cube by a reference data cube removes the background effects and results in a transmittance data cube. For this experiment, the same position cannot be selected for both the sample and reference, because the reference image must be devoid of algal cells. The difference between the two locations can (and typically does) lead to slightly different water thicknesses, generating spectral and spatial fringes with multiple frequencies.

Figure S 4(a) shows the transmittance image at one frequency (λ = 5.2μm). Figure S 4(b) shows the image at one frequency (λ = 5.2μm) when dividing the sample data cube taken at one time by the same sample data cube taken 15 minutes earlier. (sample(t1)/sample(t0)) Spatial fringes appear with higher intensity in Figure S 4(a) compared to Figure S 4(b), which is due to variation in water thickness for the sample location compared to the reference location. The low amplitude spatial fringes present in Figure S 4(b) are likely the result of slight changes in the thickness of the water layer due to non-idealities involved with the syringe pump supplying the medium.

Line scans for the transmittance and sample(t1)/sample(t0) images at three different wavelengths are shown in Figure4(c). They are all extracted from the same column of data at $y = 10.5\mu$m (highlighted with yellow in Figure S 4(a), and (b)). The former line scans are the solid lines, while the latter are dashed lines. Note that the line scans extracted from the transmittance data have larger fringes than the sample to sample comparison. Since transmittance spectra are generated from emission spectra, we show examples of emission spectra (from the pixel highlighted with red in Figure S 4(a),) from the sample and

reference data cube that are dominated by the overall emission signal from the source and the response of the optical system, including sinusoidal variations as a function of frequency (8(d)). These sinusoidal variations in the emission spectra arise from multiple internal reflections. The micro-fluidic chamber is comprised of two CVD grown sub-micrometer thick diamond windows, a thickness chosen to reduce the impact of multiple internal reflections from within the diamond windows 18 but there are still multiple internal reflections originating from the distance between the diamond window surfaces.

Appendix C

The result of the 9.75 hours of measurement for the stressed cell (habituated to 390 ppm and exposed to 5000 ppm CO_2 flow at the chamber). The result shown in Figure S 5 shows that the structure of the cells are preserved for at least 5 hours indicating that the cell is alive and responsive to the environmental changes.

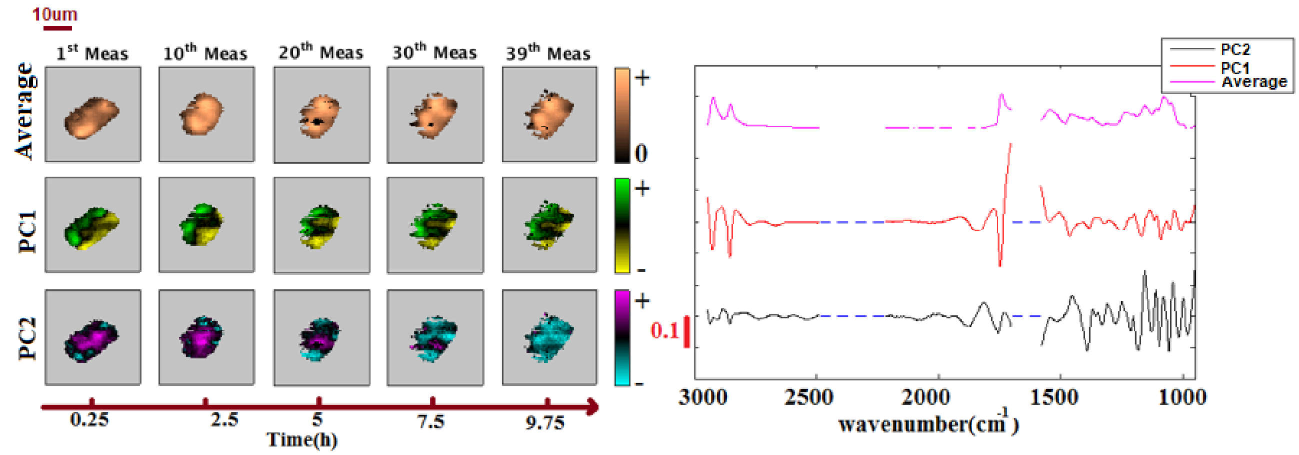

Figure S 5. Long-term response PC analysis of the Long-term response of the diatom to the stressed condition (390 ppm to 5000 ppm). The first row is the PC1 or the average, the second row is PC1 or the first variance from the average, the third row is PC2 or the second variance from the average.

Appendix D

PCA of images of three cells,"LtoH", "control", "HtoL" is included as one global PCA to evaluate the variations. The left and right separation of the cell is observed in the scores of all the cells, except the first variance score of the control cell. The second variance images of the "LtoH" have larger areas of positive sign compare to "HtoL". Larger number of cells need to be studied in order to draw a general conclusion about the distinct response of the algal cell in response to "HtoL" and "LtoH" condition. Figure

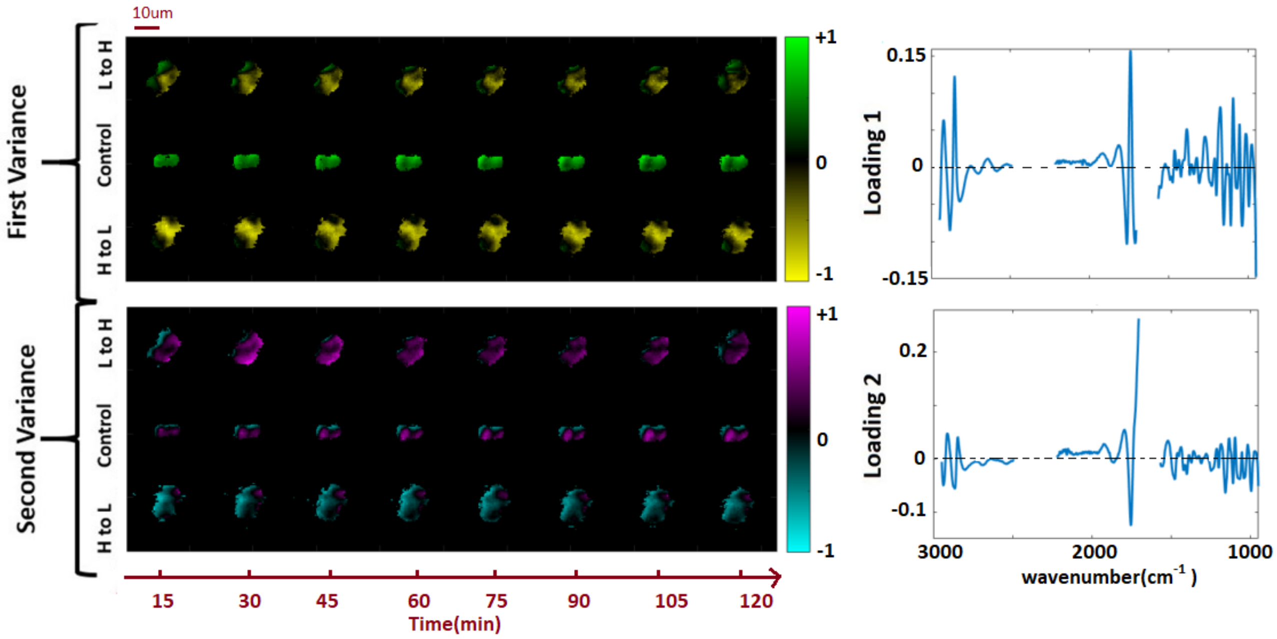

Figure S 6. PCA of the algal cells including data from all three conditions. Left: PC score images overtime; Right: PC loadings.
